# Portable Miniaturized IoT-Enabled Point-of-Care Device for Electrochemical Sensing of Zopiclone in Cocktails

**DOI:** 10.3390/bios14110557

**Published:** 2024-11-16

**Authors:** María Gabriela Mejía-Méndez, Paula C. Cifuentes-Delgado, Sergio D. Gómez, Crhistian C. Segura, Nancy Ornelas-Soto, Johann F. Osma

**Affiliations:** 1 Department of Biomedical Engineering, Universidad de los Andes, Cra. 1E No. 19a-40, Bogotá D.C. 111711, Colombia; mg.mejia@uniandes.edu.co (M.G.M.-M.); pc.cifuentes@uniandes.edu.co (P.C.C.-D.); 2 Department of Electrical and Electronic Engineering, Universidad de los Andes, Cra. 1E No. 19a-40, Bogotá D.C. 111711, Colombia; sd.gomez@uniandes.edu.co (S.D.G.); cc.segura@uniandes.edu.co (C.C.S.); 3 Laboratorio de Nanotecnología Ambiental, Escuela de Ingeniería y Ciencias, Tecnológico de Monterrey, Monterrey 64849, Mexico; ornel@tec.mx

**Keywords:** electrochemical analysis, date-rape drug detection, zopiclone, biosensor

## Abstract

This study proposes a portable and IoT-based electrochemical point-of-care sensing device for detecting zopiclone in cocktails. The system utilizes an electrochemical laccase biosensor and a potentiostat, offering a low-cost and portable device for detecting this sedative drug in cocktails. The sensor characterization experiments demonstrated the linear behavior of the oxidation and reduction currents for each of the targeted concentrations of zopiclone, enabling their detection and quantification even when mixed with an interfering substance. The proposed system could be used for the in situ analysis of cocktails, providing a valuable tool for monitoring the presence of hypnotic drugs in various social and clinical settings. The study utilized materials and reagents, including zopiclone, lab-made lemon juice, lab-made tequila, and lab-made triple sec, all prepared with reactants obtained in Bogotá, Colombia. The potentiostat used in the system was designed to manage cyclic voltammetry measurements. The electrochemical cells’ durability and longevity were also tested and characterized, with all electrodes undergoing 200 tests and their performance degradation varying according to the molecule used. The study concludes that the proposed system offers a valuable tool for detecting and monitoring pharmaceutical substances in various interfering ingredients that build up cocktails. Further research and application of this system can help address the global concern surrounding the administration of hypnotic substances to unknowing consumers through food or drinks to enable robbery and sexual assault.

## 1. Introduction

In past decades, the substances used to incapacitate individuals, often in social settings, and render them vulnerable to sexual assault have come to be known as date-rape drugs. According to [1], these drugs are named for their use in “date rape”, which refers to a form of sexual assault (drug-facilitated sexual assault or DFSA) that occurs on a date or at a social event. This kind of sexual assault occurs due to the following two circumstances: (1) when abusers secretly give drugs to incapacitate potential victims, preventing them from remembering details of the crime due to memory impairment (this corresponds with predatory or involuntary drug-facilitated sexual assault) and (2) when victims voluntarily take drugs, and the abusers exploit the resulting “stunned” state of the victim (which is referred to as voluntary or opportunistic drug-facilitated sexual assault) [2]. In terms of iDFSA (involuntary), date-rape drugs are often administered through alcoholic beverages, which can potentiate the central nervous depression generated by the drugs [3].

The potency and the speed with which date-rape drugs act make them an effective tool for ensuring that victims have impaired memory and physical control, which complicates subsequent legal and medical interventions [1]. The most common types of date-rape drugs include sedatives, hypnotics, and tranquilizers; these drugs are often colorless, tasteless, and odorless when dissolved in beverages, which allows them to be administered surreptitiously [4] and poses a significant challenge for both detection and prevention efforts. The most used substances for DFSA are alcohol, benzodiazepines (and benzodiazepine analogs), antidepressants, muscle relaxants, antihistamines, over-the-counter sleep aids, hallucinogens (such as ketamine), and opioids [5]. Nonetheless, the most used are alcohol, cocaine, and benzodiazepines [6], as well as Rohypnol (flunitrazepam), ketamine, and GHB [7].

In terms of electrochemical sensing, the three-electrode concept has matured significantly, evolving into a versatile tool in the realm of point-of-care testing (POCT) applications, thanks to advancements in technology, including the internet of things (IoT) and artificial intelligence/machine learning (AI/ML) in biomedical research. The three-electrode system typically consists of a working electrode, a reference electrode, and a counter electrode. This configuration allows for greater control over the electrochemical environment, making it easier to detect various analytes with high precision. Over recent years, developments in the miniaturization of these systems have become critical, enabling the construction of portable devices that can be used outside of traditional laboratory settings, thereby facilitating rapid diagnostics [8].

Moreover, integrating IoT technologies has further transformed the biomedical research landscape and clinical applications. Connecting devices to the internet allows data from electrochemical sensors to be transmitted in real time, allowing immediate analysis and feedback. This feature enhances patient safety and promotes timely medical intervention [9,10]. Incorporating AI and machine learning algorithms into bio-sensing platforms is another breakthrough; these technologies can improve detection accuracy, adapt to various conditions, and analyze complex data sets efficiently [11], which is essential for drug detection in social situations like those associated with DFSA.

Nowadays, only 22% of all electrochemical date-rape drug sensors are formulated to be used on beverages, while most of them [12,13] can detect these substances in bodily fluids after their consumption, which is counter-effective in terms of the prevention of said consumption [14]. Currently, most date-rape drug sensors are focused on the detection of benzodiazepines (with over 60% of sensors concentrating on these kinds of molecules) and do not report non-benzodiazepine hypnotic alternatives [14]. Despite the importance of considering benzos when designing date-rape drug sensors due to their rapid action and efficiency in sedating users, these molecules are often controlled and, in most countries, can only be accessed through psychiatric prescription, unlike zopiclone, which is an over-the-counter drug in several countries.

Zopiclone is a non-benzodiazepine hypnotic commonly denominated as a “Z-drug”, which is a popular name given to benzodiazepine pharmaceutical alternatives that are typically prescribed for the short-term treatment of insomnia despite also having anxiolytic and anticonvulsant properties. Zopiclone acts on the brain’s gamma-aminobutyric acid (GABA) receptor complex to induce sleep [15], making it practical for initiating and maintaining sleep. However, this property also makes it suitable as a date-rape drug due to its rapid onset of action and potent sedative effects. The therapeutic doses of zopiclone that are usually prescribed to patients range from 7.5 mg to 15 mg a day, which is considered a safe dosage but is not recommended for consistent use in periods over a week; the consumption of amounts over 15 mg per day or the use of this drug on periods over ten days is considered unsafe [16].

Taking the latter into account, the ability to detect the presence of zopiclone in beverages quickly and accurately is crucial, as early detection can prevent potential sexual assault and facilitate timely medical interventions. Portable detection devices offer a practical solution in various environments, from social venues to forensic laboratories. Developing such portable methods allows for the rapid screening of beverages, thereby providing an immediate response option that can enhance individual safety and judicial processes [17].

This study proposes the design of a point-of-care sensing device for detecting zopiclone in cocktails using an electrochemical laccase biosensor and a portable potentiostat, as proposed by [18]. As stated by [19], laccases are often immobilized on electrodes due to their ability to catalyze redox reactions in various substrates; the authors describe a high-redox-potential laccase obtained by computer-guided mutagenesis combined with directed evolution and immobilized on a gold electrode for electrochemical studies [19]. The novel proposed system comprises a small sensor connected to a miniaturized cyclic voltammetry electronic system for remote biosensing. It can send data via Wi-Fi, allowing it, through the integration of IoT platform technology, to alert users who are not nearby [18]. This system is a rapid, efficient, low-cost, and portable method for the detection of zopiclone in beverages, aiming to contribute to the broader effort to combat drug-facilitated crimes. To the best of our knowledge, no similar electrochemical biosensors have been made to facilitate the detection of zopiclone in cocktails.

## 2. Materials and Methods

### 2.1. Reagents

All materials were used as received without further purification. Pills containing 7.5 mg of zopiclone were acquired from Humax Pharmaceutical (Bogotá, Colombia). Positive controls were tested using zopiclone aliquots ranging from 77.2 mM to 205.8 mM, in which the shredded pill was diluted in Type II water. All of the aliquots used as positive controls constitute lethal doses, considering that, according to [16], the average dose of zopiclone found in victims who were killed was 53.25 mg; if diluted in 50 mL, this implies that the minimum lethal dose would have a molar concentration of 2.7 mM. Furthermore, three interfering substances were prepared to emulate lemon juice, Tequila, and TripleSec, which make up Margaritas. Initially, emulated lemon juice (ELJ) was prepared using Milli-Q (Type II) water, 3.7% (*w*/*v*) citric acid [20], and 2.6% (*w*/*v*) sugar [21] at a 1:1 mixture of glucose and sucrose. Furthermore, the emulated Tequila (ET) used in these experiments corresponds to a 50% (*v*/*v*) ethanol [22,23] solution in Milli-Q (Type II water). Finally, the emulated TripleSec (ETS) corresponds to a solution composed of 30% (*v*/*v*) ethanol [24] and 37% [25] of a half-and-half sucrose and glucose mixture. Negative controls were tested using each of the previously mentioned interfering substances.

### 2.2. Potentiostat Design and Electrode Design

The potentiostat previously designed by our group [18] used in this system was built using an MSP430G2553 microcontroller from Texas Instruments (Dallas, TX, USA) to manage cyclic voltammetry (CV) measurements. The system creates a triangular stepped signal with a fixed frequency composed of counter (CE) and reference (RE) electrodes and which measures the resulting current at the working electrode (WE). This potentiostat delivers CV measurements with high precision and accuracy, capable of detecting electrochemical reactions with a resolution of 10 nA and sensitivity of 3.3 nA. Its low-cost design, requiring only a microcontroller and a few electronic components, makes it an attractive option for portable electrochemical biosensing applications. The compact device, approximately 5.5 mm × 6.5 mm and weighing around 20 g [18], offers separate USB–UART connectivity and is compatible with commercial modules. Additionally, its microcontroller software supports multiple electrochemical techniques, and the desktop software can now connect to a remote web server [18]. Compared with an AUTOLAB PGSTAT 101 commercial system (Utrecht, The Netherlands), tests on a dummy cell demonstrated the potentiostat’s effectiveness as a low-cost alternative, offering standardized, comparable, and repeatable measurements. Further tests showed that the developed potentiostat produced reduction and oxidation peaks like those of the commercial equipment, with minor discrepancies attributable to the tolerance of electronic components and low-pass filters, which can be adjusted through software amplification options [26]. Therefore, this potentiostat presents an excellent alternative for conducting electrochemical tests, such as that required for zopiclone detection.

All of the electrochemical cells used in this system are based on the design by [18], with each cell consisting of a three-electrode configuration: the working electrode (WE), the counter electrode (CE), and the reference electrode (RE). In the present work, Eagle version 9.6 by Autodesk (San Francisco, CA, USA) was used to design the cells, with the sample holder area measuring approximately 380 mm^2^. The WE, CE, and RE areas were 78.5 mm^2^, 35 mm^2^, and 5 mm^2^, respectively. The PCB fabrication process was followed to manufacture the electrodes, starting with a sheet of FR4 with a thin layer of copper (approximately 70 µm) covered with a photosensitive polymeric layer. After exposing the desired pattern to light and removing the unexposed polymer with a stripper, the copper was removed using wet chemical etching. A small tin layer was applied to prevent electrode oxidation. Finally, a UV mask was placed to define the sample area and protect the electrodes. A detailed cost estimation for the proposed device was made, calculating a total production cost of USD 26.92, the details of this estimation can be found in Supplementary Material S3.

### 2.3. Production and Purification of Laccase

The laccase enzyme was produced using *P. sanguineus* CS43, which was grown on a tomato medium using the prior methodology explained by [27]. Mycelia from the culture supernatant was filtered through a serially occupying pair of filters with 0.5 mm and 0.2 mm pore sizes. After this stage, ultrafiltration was carried out using a ten kDa cut-off membrane. The execution of these stages resulted in procuring two isoforms of the laccase enzyme, Lac I and Lac II, which harbored a high degree of similarity in their amino acid sequences (91%). These isoforms displayed a noteworthy heat stability, which was demonstrated by their capacity to maintain optimal activity at temperatures up to 50 °C and 60 °C. In practice, the half-life of Lac I at these temperatures was established to be 277.7 h and 18 h and Lac II’s to be 35.8 h and 2.25 h, respectively. The molecular weights of the respective isoforms were determined to be 68 kDa and 66 kDa [27].

### 2.4. FTIR Characterization

The present study aimed to analyze changes in the chemical composition of the working electrode surface before and after their respective use via Fourier transform infrared (FTIR) spectroscopy. The electrochemical laccase biosensors were characterized using a Bruker (Billerica, MA, USA) ALPHA FTIR Eco-ATR spectrophotometer, which provided a spectral measurement range of 500–4000 cm^−1^ with a resolution of 2 cm^−1^. Before the data analysis, data were subjected to five moving average filters with a window size of four. The Sigma Aldrich IR Table [28] determined the IR spectrum. Only the working electrode of each sensor was characterized as the other electrode that underwent modification (reference sensor) has no chemical bonds, and, therefore, no results would be obtained in the FTIR characterization [29].

### 2.5. SEM and EDS Characterization

The surfaces of used and unused working electrodes were examined to gain insight into their topography and composition changes following multiple uses for the LCA. Scanning electron microscopy (SEM) and energy-dispersive X-ray spectroscopy (EDS) were employed. The SEM analysis was conducted using a TESCAN (Brno, Czech Republic) LYRA3 FIB-SEM instrument with an accelerating voltage of 10 kV. EDS was run during SEM imaging to determine the elemental analysis and mapping of the electrodes.

### 2.6. Sensor Design

This point-of-care sensing device comprises a copper counter electrode, an Ag/AgCl reference electrode, and a working electrode that underwent a surface functionalization with APTES, glutaraldehyde, and laccase and, therefore, became an electrochemical laccase biosensor. These biosensors were used to perform voltametric tests on zopiclone alone, as well as when mixed with each of the interfering substances, which also generated reduction and oxidation currents. Each of these substances was found to have a more linear behavior in terms of oxidation current; additionally, as each of them has a different oxidation voltage, oxidation currents were selected to identify each of these substances.

The deposit of Ag/AgCl was made by following the method used by [28], in which the reference electrodes were covered in liquid silver and were later heated for 30 min (at 45 °C). Once removed from the heating plate, 1 μL of HCL 0.1 M was added directly onto the (now dry) silver and was left for 30 min. Finally, it was removed with a micropipette and washed with type II water. Electrode deposits that use silver and HCl to create AgCl are commonly used in electrochemical applications due to their usability as a molecule immobilization platform [30].

In addition to this, the electrodes were subjected to laccase immobilization on their working electrode using APTES, glutaraldehyde, and laccase, following the process used by [28], in which the working electrode was initially cleaned with type II water, which was then removed. Furthermore, the electrode was silanized using 8 μL of 2% *v*/*v* 3-amino propyl tri ethoxy silane (APTES) to activate the surface, which was left on the electrode for 30 min. Afterward, 8 μL of 2% *v*/*v* glutaraldehyde was added and left for 30 min to act as a crosslinker and to immobilize the laccase onto the nanoparticles. Finally, 8 μL of 2000 U/L laccase was applied and left to dry overnight. The immobilized laccase showed high catalytic activity [31].

### 2.7. Point-of-Care Sensing Device Design and Characterization

**Figure 1:**
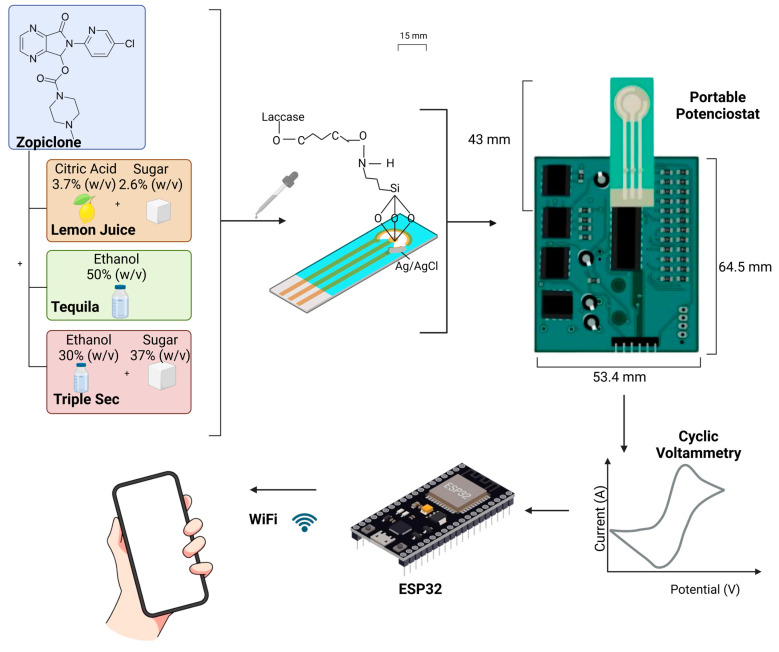
The steps of the methodology implemented for the laccase biosensor characterizations with each zopiclone concentration. First, control measurements were made using only zopiclone. Furthermore, each concentration was mixed with the emulated interfering substances (ELJ, ET, and ETS); in all the previously mentioned circumstances, measurements were made using a laccase biosensor and a portable potentiostat which, using Wi-Fi and IoT platform technology, sent the measured data to a phone. Diagram built using BioRender (Toronto, ON, Canada) [33].

The potentiostat used in this system was characterized by dummy cells and compared with the AUTOLAB and PalmSens commercial systems. The characterization was performed using analog components, with the only digital part being the microcontroller. As can be seen in Figure 1, the system was connected to an IoT web platform, which allowed connection to the ESP-WROOM-32 microcontroller (Espressif, Shanghai, China) using the UART protocol, managing communication commands from the cloud and sending them to the microcontroller. Furthermore, the generated data were saved and made available to the user, enabling remote control to create new readings. The dummy cells were used to test the output of both systems against a pure resistive cell, the production of both potentiostats with a Randles cell, and the response with a redox system [18].

### 2.8. Point-of-Care Sensing Device Testing with Zopiclone

When the sensor encounters the analyte (in this case, zopiclone, which may or may not be mixed with the lab-made interfering substances), the bioreceptor (in this case, the laccase enzyme) generates a biochemical reaction, which, at the same time, creates a chemical signal; furthermore, said chemical signal is converted into an electrical signal by the potentiometric transducer. Then, the signal is conditioned and processed by the microprocessor unit. Finally, the output signal is sent to the ESP-32 controller, which uploads it to an IoT platform through Wi-Fi, which allows it to be accessed through any Wi-Fi-connected device [32]. This is displayed in Figure 1, which showcases a step-by-step process that is undertaken for each compound using the electrochemical laccase biosensor, in conjunction with a potentiostat, to conduct a cyclic voltammetry analysis using maximum voltage (V max), minimum voltage (V min), and slope.

**Figure 2:**
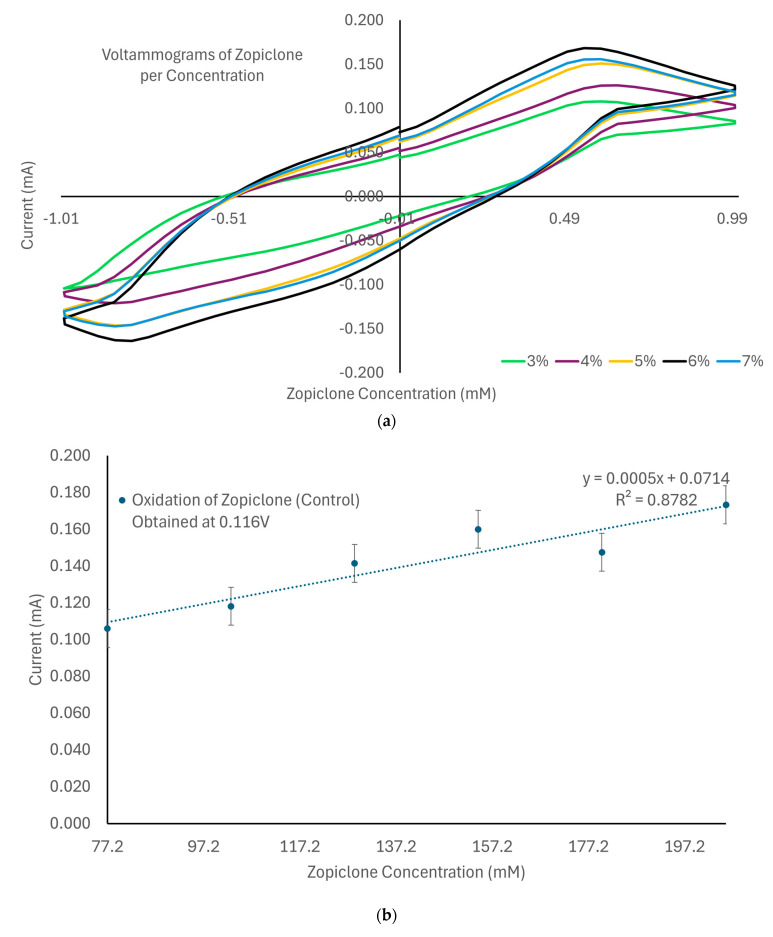

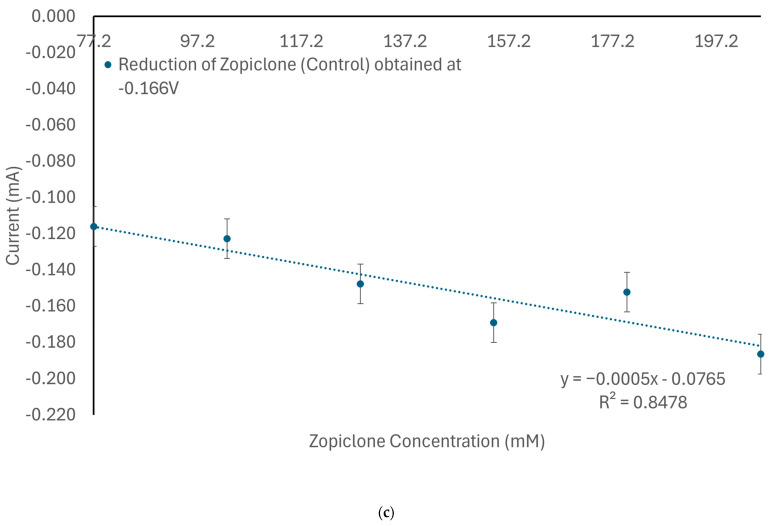
(**a**) Voltammogram of zopiclone per concentration for concentrations from 3 to 7% (*w*/*v*). (**b**) Curve suggesting the existence of a linear dependence between oxidation current and concentration of zopiclone. (**c**) Curve suggesting existing linearity between reduction current and concentration of zopiclone.

The laccase biosensor’s characterization with various concentrations of zopiclone was performed by mixing solutions ranging from 77.2 mM to 205.8 mM (77,158.91 µM to 205,757.08 µM) using shredded pills with Milli-Q water. The resulting control solutions were then subjected to cyclic voltammetry measurements using electrodes connected to the potentiostat. Each sample was subjected to an initial voltage of 0 (V), a scan rate of 0.050 (V/s) for 20 cycles, and two replicates for 40 measurements per sample. Each cycle’s first and second vertices were set at 1.0 (V) and −1.0 (V), respectively. The collected data included oxidation and reduction points for each cycle. Furthermore, these values were averaged and compared, seeking to find a linear behavior between currents and concentrations. This allowed us to select oxidation current as the parameter with the best linearity. The initial results can be seen in Figure 2, where an average voltammogram of zopiclone according to its concentration, as well as curves for its oxidation and reduction currents, demonstrate the dependence between these variables and the concentration of zopiclone. Lastly, the limit of detection (LOD) for each of the interfering substances, as well as the positive controls, was calculated using the formula LOD=3m+b [34], where m and b are factors in the trendline equation y=mx+b.

Furthermore, each zopiclone solution was tested when mixed at a 1:2 rate with the previously mentioned interfering substances (ELJ, ET and ETS) using the same parameters mentioned for the control. The mixtures were then tested using a laccase biosensor and a portable potentiostat, which generated electrochemical cyclic voltammetry readings and were sent to a nearby cell phone through Wi-Fi using IoT platform technology.

### 2.9. Cycle of Operations

The laccase biosensors were tested using the app developed by our group [18] for 200 repeated measurements using zopiclone at a 25.7 mM concentration to test the biosensor’s half-life. Additionally, the electrodes were cleaned with Milli-Q water in between repetitions. Initial parameters for these measurements were a start point of 0 (V); a scan rate of 0.005 (V/s); first and second vertices at 1.0 (V) and −1.0 (V), correspondingly; and ten cycles of readings per measurement.

## 3. Results and Discussion

Original data can be found in the Supplementary Material S1 and S2.

### 3.1. FTIR Characterization Results

**Figure 3:**
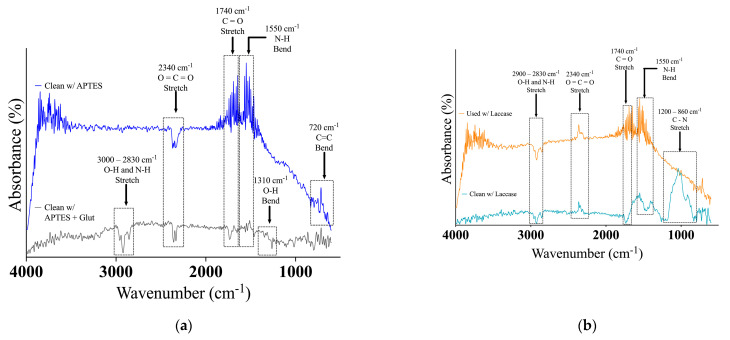
FTIR analysis of (**a**) unused APTES only and APTES + glut electrodes and of (**b**) unused and used laccase-immobilized electrodes.

Upon analyzing the spectra in Figure 3a, it was determined that the unused APTES electrode displayed a band at 720 cm^−1^, indicating C=C bending vibrations. This band is associated with a cis-disubstituted alkene group and is believed to result from the presence of the alkene group in the APTES chemical structure [22]. Furthermore, FTIR analysis revealed that both electrodes exhibited similar vibrations linked to carboxylic acid groups. Specifically, a band at 1740 cm^−1^, corresponding to carbonyl stretch C=O from a dimerized carboxylic acid, was detected. Additionally, a band at 1550 cm^−1^, related to N-H bending, was observed on both spectra, indicating the presence of amide bonds. These bonds may have formed before laccase immobilization or could be residues from being stored close to laccase-immobilized electrodes. Lastly, a significant band at 2340 cm^−1^, associated with O=C=O stretching vibration, was observed on both spectra. According to [30], this can be attributed to band overlap and random noise.

However, a notable distinction was observed in Figure 3a between the APTES and APTES + glut electrodes: the unused APTES + glutaraldehyde electrode displayed a band at 1310 cm^−1^, corresponding to an O-H bending vibration.

The electrode surface exhibited the presence of an aldehyde chemical group because of the glutaraldehyde reagent. Additionally, the spectrum revealed the existence of O-H and N-H stretching bands at around 2950 cm^−1^, which were associated with O-H and N-H stretching combined. This could be attributed to hydrogen-bonded dimers of carboxylic acid groups (amino groups) on the APTES + glutaraldehyde electrode. These bands confirm the binding interaction necessary for laccase immobilization.

In contrast, the FTIR analysis in Figure 3b clearly illustrates noticeable differences caused by laccase immobilization, indicating its success. The unused and used laccase-immobilized electrodes exhibit similar vibrations corresponding to specific chemical groups. However, the latter also displays signs of wear on the electrode surface. For example, peaks below 890 cm^−1^ in the blue spectrum, which are believed to indicate C-H bending vibrations, suggest the presence of residual organic compounds from prior usage. The size of the peak is directly related to the quantity of laccase that is immobilized on the electrode. This suggests that a substantial amount of laccase is present and active on the electrode.

Additionally, both spectrums display a prominent peak at 1550 cm^−1^, representing the N-H bond’s bending vibrations. This supports the successful immobilization of laccase using APTES functionalization, which contains amino groups in its structure [29]. Another peak at 1740 cm^−1^ indicates the presence of carbonyl groups, likely due to organic compounds like laccase proteins [29]. The band ranging from 2830 to 3000 cm^−1^ corresponds to the distinctive traits of free laccase, including O-H and N-H stretching vibrations inherent to the enzyme’s structure and C-H stretching vibrations caused by organic groups [30]. Lastly, the unused electrode illustrated in Figure 3b displays an O=C=O peak similar to the random noise observed in Figure 3a.

### 3.2. SEM and EDS Characterizations

**Figure 4:**
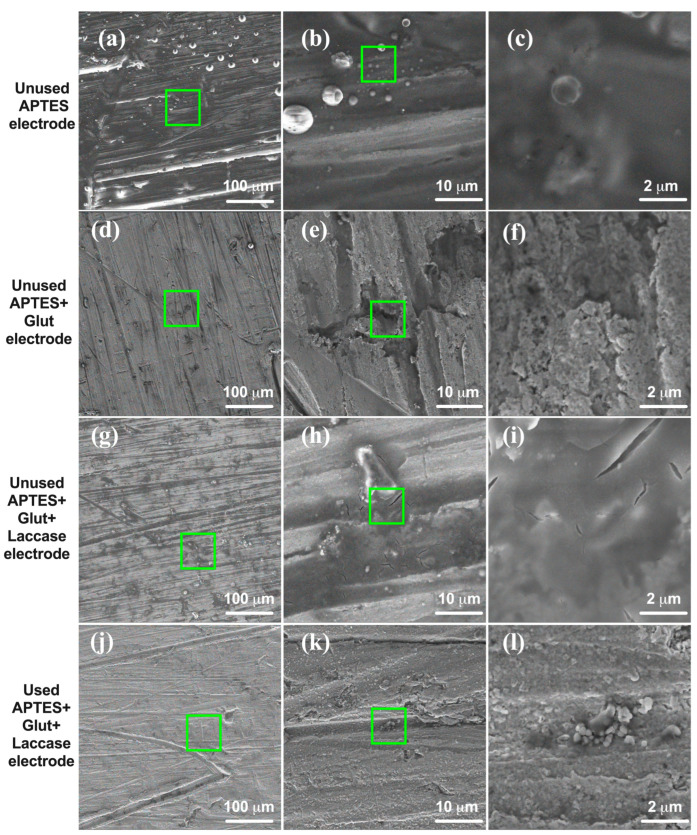
SEM digital images at 1 kX (**left**), 10 kX (**center**), and 40 kX (**right**) magnifications. Ten kV of accelerating voltage was applied to (**a**–**i**) unused electrodes and (**j**–**l**) used electrodes. The green square indicates where the magnifications were made.

SEM was employed to analyze the physical characteristics of the electrode surface and to compare any changes observed after repeated use. The process of laccase immobilization was carefully tracked by examining the electrode surface at different stages: with APTES alone, with APTES and glutaraldehyde, and, finally, with immobilized laccase. When the unused APTES electrode’s working segment was magnified, it appeared somewhat irregular. It had droplet-like shapes, possibly due to the manufacturing process and which might be associated with the used reactant (Figure 4b). This irregularity remained essentially unchanged after APTES treatment. Similarly, irregularities were still present in the APTES + glut and laccase-immobilized electrodes (Figure 4d and Figure 4g, respectively), indicating that the laccase layer did not significantly affect the electrode’s morphology. However, Figure 4 depicts the presence of the immobilized laccase enzymes.

In contrast, the SEM image of the used laccase electrode demonstrated noticeable signs of wear and tear on the working segment (Figure 4j), which can be attributed to repeated use. Furthermore, it was evident that the immobilized laccase had detached from the electrode surface.

The study examined the electrode’s morphology and integrity, specifically its immobilization process and reliability. SEM analysis determined that the electrode’s basic structure remains intact even after wear and tear, indicating that the immobilization process has minimal impact on its overall morphology.

**Figure 5:**
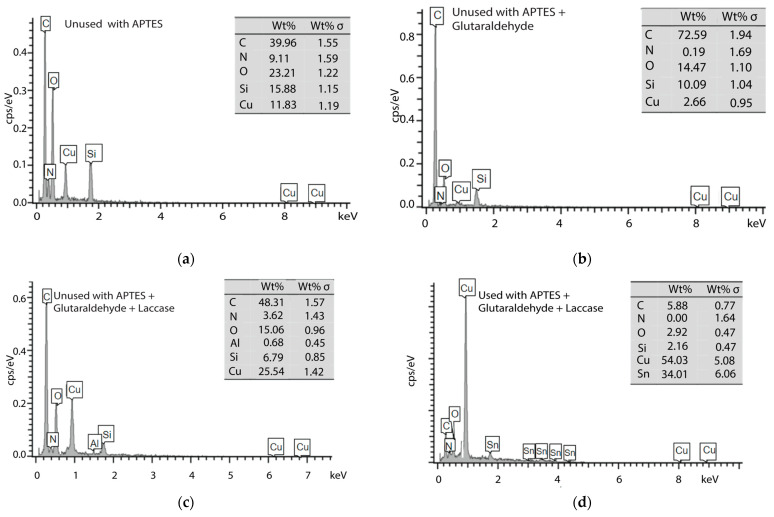
EDS graphic characterization of (**a**–**c**) unused electrodes and (**d**) used electrodes.

To analyze the electrodes at an atomic level, EDS was used to generate graphics depicting the elements present on the surface of the working segment. Figure 5a displays the spectrum of the unused APTES electrode, revealing a composition primarily consisting of carbon, nitrogen, silicon, and oxygen. This composition is a result of the APTES coating on the working electrode, which contains ethoxy groups and an amine group in its structure [31]. Additionally, small amounts of silicon and copper are present. The silicon could be attributed to the interaction and storage of the unused electrodes with the used electrode. At the same time, the copper may be a result of the electrode manufacturing process, which includes an electroplating step to minimize corrosion.

On the other hand, the unused APTES + glut and laccase-immobilized electrodes exhibit the same elemental composition as the unused APTES electrode (Figure 5b,c), with the addition of trace amounts of aluminum. This aluminum could be linked to impurities in the added glutaraldehyde.

The elemental composition of the laccase electrode used in the study (Figure 5d) was similar to that of an unused laccase electrode, except for the absence of aluminum and tin. The main elements in this composition were copper and tin, indicating some degradation of the reagents used for laccase immobilization. However, there were also carbon, nitrogen, and oxygen traces, suggesting that the electrode could protect the copper from deterioration. Additionally, elements like carbon, silicon, and oxygen align with the electrode’s purpose of analyzing organic compounds like pharmaceutical contaminants.

### 3.3. Potentiostat Characterization

The potentiostat could perform electrochemical measurements with a similar accuracy and precision as commercial systems. Specifically, the comparison demonstrated that the in-house potentiostat responded similarly to the commercial systems regarding the output voltage and current and the shape of the curves. These results suggest that the in-house potentiostat implemented for this system is a viable alternative to commercial systems for electrochemical measurements [26].

The analysis of each of the previously mentioned substances was carried out according to the methodology described earlier, resulting in both reduction and oxidation currents being obtained for each except acetaminophen, for which only oxidation currents were obtained. The current with the best linearity was carefully chosen, as seen afterward; for this, a threshold of 0.80 linearity coefficient was set to ensure reliable results.

### 3.4. Laccase Biosensor Characterization with Pharmacological Substances

**Figure 6:**
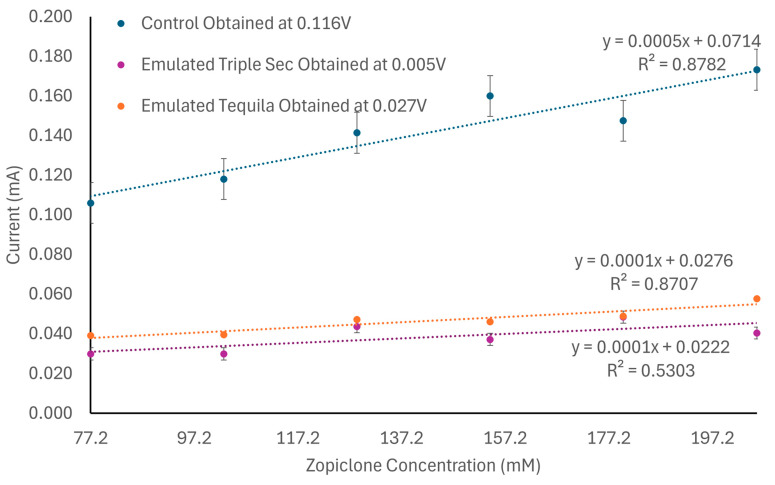
Line graph of the results obtained for the tests performed on zopiclone when mixed with no other substance (which oxidized at 0.116 V), when combined with ETS (which achieved its oxidation at 0.005 V), and when mixed with ET (which oxidized at 0.027 V).

When measuring the different concentrations of zopiclone, the analysis demonstrated that this substance achieved its peak oxidation current at 0.116 V and maximized its linearity when analyzing concentrations between 77.2 mM and 205.8 mM. When measuring concentrations above this, the laccase biosensor quickly saturated; meanwhile, it was undersaturated when measuring concentrations below this range, leading to inconclusive results. As shown in Figure 6, the data obtained for these concentrations indicated that, when in oxidation, the zopiclone control behaves following the formula y = 0.0005x + 0.714 and can achieve an R^2^ of 0.8782, which implies a sensibility of 0.0005 mA/mM. Furthermore, when testing the interference of ELJ, the laccase biosensor, unfortunately, became saturated, independently of the measured concentration of zopiclone. Therefore, its interference will not be reported.

On the other hand, when testing the interference of ET within the previously mentioned concentration range, oxidation was achieved at 0.005 V. The behavior of this interfering substance is described by the equation y = 0.0001x + 0.0276, which implies a sensibility of 0.0001 mA/mM and a linear coefficient of 87.04%. Therefore, this interfering substance reached only 27% of the original sensibility, which can occur due to the high volatility of alcohol, which leads to a quick reduction of the analyzed amount of sample. The oxidation points obtained for zopiclone when using this interference are reported in Figure 6.

TripleSec, as an interfering substance within the previously mentioned concentration range, oxidized at 0.027 V. As can be seen in Figure 5, the behaviors for this substance are described by the trendline equation y = 0.0001x + 0.0222; therefore, a sensibility of 0.0001 mA/mM was obtained, as well as a linearity of 0.5307. In this case, both the sensibility (23% of the original) and the linearity were reduced, which can be due to the presence of alcohol within the solution as well as the increased density of the solution (in comparison with the control) due to the high amount of sugar within it.

Despite the changes in sensitivity and linearity obtained when measuring the interfering substances, the tendency of the oxidation of zopiclone was preserved regardless of the use of interfering substances, as seen in Figure 6. Table 1 summarizes the data obtained when analyzing zopiclone (converted into molarity) with each interfering substance.

### 3.5. Cycle of Operations Results

**Figure 7:**
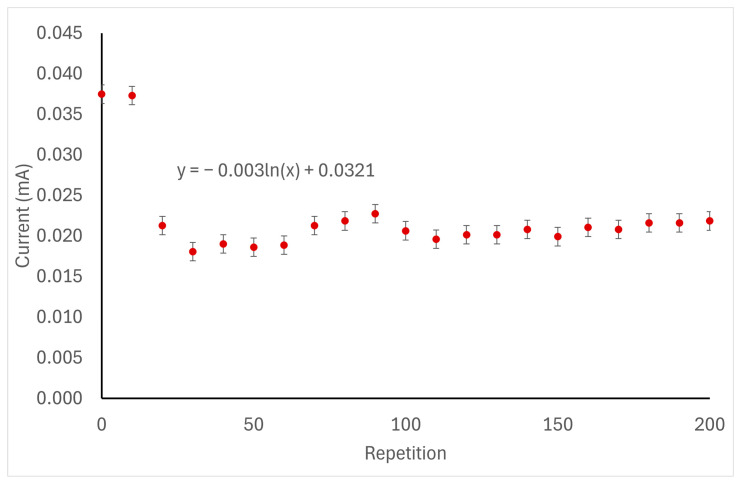
Oxidation current of 25.7 mM of zopiclone when using the laccase biosensor 200 times.

When measuring the cycle of operations of the laccase biosensor using zopiclone, the substance achieved its oxidation point at 0.116 V. In the first repetition, the oxidation current identified was 0.037 mA. After 200 uses, this measurement decreased to 0.022 mA, representing a 40.54% reduction in the measured oxidation current relative to the initial measurement. The degradation of this laccase biosensor is described by the function y = −0.003ln(x) + 0.0321. In the performed measurements, this laccase biosensor did not reach 50% of its capacity (d50), which would have been at 0.0185 mA. Nevertheless, a considerable decrease in performance can be identified after the 20 uses, which can be attributed to the loss of laccase or a decline in its enzymatic activity. Only the wholly assembled sensor was tested, and no tests were performed on its components. This behavior can be seen in Figure 7.

### 3.6. Discussion

To the best of our knowledge, there is a low quantity of studies related to detecting zopiclone while accounting for the interfering substances that may be present in cocktails. Therefore, the vast majority of the documents cited in this section are not related to the detection of zopiclone in cocktails but rather to the identification of other date-rape drugs, including benzodiazepines, such as flunitrazepam [35], diazepam [3,36,37], midazolam [37,38,39,40], and lorazepam [41], in cocktails, as well as ketamine [42], and gamma-hydroxybutyric acid (GHB) [43,44]. Additionally, other studies regarding the detection of tobacco [45], the cannabinoid designer drug ADB-BUTINACA [46], and zopiclone [47] in mediums other than cocktails have been included for comparison.

Several sensors have been developed to primarily detect benzodiazepines during testing with interfering substances. For example, the authors in [35] evaluated a screen-printed carbon electrode with Ag/AgCl on the reference electrode, which has analogous reactants to the evaluated sensor and is used for the detection of flunitrazepam, a well-known benzodiazepine. In another study by [36], a silver thread sensor was designed and implemented to identify diazepam; this sensor showed a higher sensitivity than the one evaluated in the present work, which can be attributed to the silver and to the manufacturing process carried out for the sensor. Furthermore, the paper by [3] was able to detect diazepam when mixed with cocktail interferents and serum using a carbon paste, glassy carbon powder, multiwalled carbon nanotube electrode (CPE/GCPE/MWCNTs), managing a limit of detection of 0.33 μM and a sensitivity of 0.21 μA/μM [3]. Another relevant study, in which the authors managed to detect two different rape drugs (diazepam and midazolam), tested a laser-scribed graphene sensor with the use of interfering substances [37]. In addition, other studies have managed to detect benzodiazepines using materials such as graphite on aluminum sandpaper [38], boron-doped diamond electrode (BDDE) [39], or Au-NPs@Silica modified carbon paste electrode [40], which were used to detect midazolam using different techniques and interfering substances and which obtained limits of detection as low as 6.1 μM, 0.46 μM, and 0.0224 μM, respectively [38,39,40]. Lastly, the study by [41] identified lorazepam using SWV, achieving a sensitivity of 0.371 μA/μM and a detection limit of 50 nM [41].

Other relevant rape drugs are ketamine and GHB, which have also been evaluated in various studies. One of these, ketamine, was detected using a hybrid sensor of zeolite nanoflakes and graphene oxide nanocrystals (Zeo-GO), achieving the lowest limit of detection (LOD) among all investigated sensors [42]. Conversely, a GHB detection electrode modified with glassy carbon, platinum nanoparticles, and polyvinyl alcohol, which also used interfering substances, had a slightly lower LOD than the sensor evaluated in this study [43]. Lastly, in [44], a paper-based cell-free biosensor was proposed for the detection of GHB using BlcR from *Agrobacterium tumefaciens* and *Shigella flexneri* MerR transcriptional activator; this sensor uses fluorescence as an indicator of the presence of this molecule and does not consider interfering substance nor have an IoT connection.

Moreover, studies on the detection of non-date-rape drugs that also have psychoactive effects have also been performed. On the one hand, the study by [45] implements ferrocene-functionalized multiwalled carbon nanotubes (FC-MWCNTs) to detect tobacco through the implementation of cyclic voltammetry and electrochemical impedance spectroscopy (EIS), obtaining an LOD of 4.25 μM. On the other hand, the work by [46] was able to detect ADB-BUTINCA, a famous designer cannabinoid common in Europe, by using a modification-free boron-doped diamond electrode in an aqueous electrolyte solution, managing to obtain a sensitivity of 0.20 μA/μM and an LOD of 0.83 μ mol dm^−3^ [46]. Lastly, the paper by [47] presents the electrochemical detection of zopiclone using an electrochemically reduced graphene oxide electrode (GC/rGO) using square wave adsorptive stripping voltammetry, managing to obtain a sensitivity of 0.164 μA/μg L as well as a limit of detection of 2.14 μg/L [47].

Table 2 summarizes the main features of the different alternatives used for the detection of drugs. When comparing the biosensor we created to the other options found, it is evident that our point-of-care sensing device is the only one that implements IoT among those analyzed in Table 2. Additionally, only our device and that of [47] include biosensors. As can be seen in Table 2, most of the devices described use electrochemistry, specifically techniques such as cyclic voltammetry (CV) or square wave voltammetry (SWV), and only that of [47] uses fluorescence. Though the LOD obtained by our system is not the lowest, it is among the most sensitive, as referenced in Table 2. Finally, it is essential to highlight that our solution is formulated as a point-of-care sensing device and seeks to prevent date-rape drug-related attacks in public areas. Therefore, its portability and the use of IoT technology show improvements toward total integration.

The spectroscopic characterization of the unused and used electrodes had some critical findings. Firstly, spectra from Figure 4a indicated potential contamination of the unused electrode with residues from the used (naked and Ag/AgCl) electrodes, as most of the observed chemical groups were attributed to signal noise or remnants from measured reagents. However, the influence of laccase immobilization can be seen in Figure 4b, in which successful immobilization is indicated by the distinctive peaks associated with C=O stretching (1725 cm^−1^) and N-H bending (1580 cm^−1^) of the protein bonds, as reported by [48]. This finding is further supported by the presence of the peptide linkage vibration at 1250 cm^−1^ resulting from the usage of the functionalization reagents, as observed in the studies conducted by [48].

Additionally, the EDS presented in Figure 5 corroborates the presence of well-manufactured copper layers on all three electrodes, along with traces of measured organic substances, demonstrating the unused electrode’s contamination. Furthermore, the electrode with laccase immobilization exhibited a higher abundance of the organic elements associated with the laccase structure, as described in ref. [48].

The morphological and elemental assessments helped evaluate the durability of the electrodes. Figure 4 reveals some irregularities attributed to the electrode manufacturing process described by [18]. However, its overall structure remained largely unaltered after multiple uses, demonstrating the electrodes’ endurance. However, laccase immobilization further improved this durability and reliability, as Figure 4e shows minimal wear in some localized regions while maintaining credible and consistent measurements. Moreover, the presence of this protein did not change the analyzed morphology, which is consistent with the findings reported [49].

Finally, as was proven in Figure 7, even though the laccase biosensor used in the present experiment does not have a 50% reduction in sensitivity within its first 200 uses, its sensitivity is greatly reduced after use 20. Therefore, it is recommended that this sensor is used 20 times and exchanged after that.

## 4. Conclusions

The proposed system introduces a portable, IoT-based solution for detecting zopiclone in cocktails, which can significantly enhance the safety and efficiency of point-of-care sensing devices aimed at preventing the administration of hypnotic substances to unsuspecting consumers. Experimental results demonstrate that the laccase biosensor can detect zopiclone in concentrations ranging from 77.2 mM to 205.8 mM at 0.116 V, even when mixed with lab-manufactured ET and ETS, though the oxidation current shifts to 0.005 V and 0.027 V, respectively; however, due to rapid electrode saturation, zopiclone detection was not achievable when mixed with ELJ. This sensor was able to detect the presence of zopiclone in concentrations above therapeutic and lethal doses [16].

While the laccase-modified copper electrode with Ag/AgCl reference does not outperform all other sensors in terms of sensitivity or the lowest limit of detection, its unique configuration as a biosensor—featuring immobilized laccase and Ag/AgCl—distinguishes it from others. This sensor used laccase due to its oxidative capacity, with phenol-like molecules that have previously generated an interaction with pharmaceutical substances; nonetheless, in this case the only pharmaceutical substance that was tested was zopiclone. The added benefit of IoT connectivity enhances its portability and functionality, something that none of the other sensors provide. Moreover, its capability to detect zopiclone, a frequently accessible, over-the-counter drug in various regions, underscores its potential as an innovative solution in the detection of substances used in drug-facilitated crimes.

## Figures and Tables

**Table 1:** Indicators obtained for all substances.

Interfering Substance	Concentration Range (mM)	Oxidation Voltage (V)	Trendline Equation	Sensibility (mA/mM)	Limit of Detection (LOD) (mM)	Linear Correlation
None (control)	77.2 to 205.8	0.116	y = 0.0005x + 0.0714	0.0005	0.0729	0.8782
Emulated tequila (ET)	77.2 to 205.8	0.005	y= 0.0001x + 0.0276	0.0001	0.0279	0.8704
Emulated triple sec (ETS)	77.2 to 205.8	0.027	y= 0.0001x + 0.0222	0.0001	0.0225	0.5307

**Table 2:** Comparison of the different alternatives used in the detection of drugs.

Sensor Materials	Biosensor (Yes/No)	Use of Interfering Substances (Yes/No)	IoT (Yes/No)	Drug	Detection Technique	Sensitivity	LOD	Ref.
Carbon-screen printed and Ag/AgCl reference	No	Yes	No	Flunitrazepam	CV	0.142 μA/ μM	1.8–14.6 μM	[35]
Silver thread	No	Yes	No	Diazepam	EIS	2.46867 ΩL/mg	Not reported	[36]
CPE/GCPE/MWCNTs	No	Yes	No	Diazepam	SWV	0.21 μA/μM	0.33 μM	[3]
Laser-scribed graphene	No	Yes	No	Diazepam and Midazolam	SWV	0.20 μA/ μM	0.66 μM	[37]
Graphite on aluminum sandpaper	No	Yes	No	Midazolam	SWV	Not reported	6.1 μM	[38]
Boron-doped diamond electrode (BDDE)	No	Yes	No	Midazolam	CV + SWV	Not reported	0.46 μM	[39]
Au-NPs@Silica modified carbon paste electrode	No	No	No	Midazolam	DPV	0.0034 (No units reported)	2.24 × 10^−8^ M	[40]
NiO/ SWCNT-modified carbon paste electrode	No	No	No	Lorazepam	SWV	0.371 μA/μM	50 nM	[41]
Zeolite nanoflakes and graphene-oxide nanocrystals (Zeo-GO)	No	Yes	No	Ketamine	CV	Not reported	1 nM	[42]
Glassy carbon/platinum nanoparticles/polyvinyl alcohol modified electrode (GC/PtNPs/PVA)	No	Yes	No	GHB	CV+CA	Not reported	0.872 mM	[43]
Paper cell-free biosensor based on the BlcR from *Agrobacterium tumefaciens* and *Shigella flexneri MerR* transcriptional activator	Yes	No	No	GHB	Does not apply fluorescence sensor	0.2%	0.3%	[44]
Ferrocene-functionalized multiwalled carbon nanotubes (FC-MWCNTs	No	No	No	Tobacco	CV, EIS	Not reported	4.25 μM	[45]
Modification-free boron-doped diamond electrode in an aqueous electrolyte solution	No	No	No	ADB-BUTINCA	SWV	0.20 μA/μM	0.83 μ mol dm^−3^	[46]
GC/rGO	No	No	No	Zopiclone	SWAdSV	0.164 μA/μg L	2.14 μg/L	[47]
Immobilized laccase copper electrode with Ag/AgCl reference	Yes	Yes	Yes	Zopiclone	CV	1.266 μA/mM	0.220 μM	This work

## Data Availability

The raw data supporting the conclusions of this article will be made available by the authors on request.

## Supplementary Materials

The following supporting information can be downloaded at: https://www.mdpi.com/article/10.3390/bios14110557/s1.
